# Changes in Onset of Vegetation Growth on Svalbard, 2000–2020

**DOI:** 10.3390/rs14246346

**Published:** 2022-12-15

**Authors:** Stein Rune Karlsen, Arve Elvebakk, Hans Tømmervik, Santiago Belda, Laura Stendardi

**Affiliations:** 1 NORCE Norwegian Research Centre AS, P.O. Box 6434, 9294 Tromsø, Norway; 2 Noregs Arktiske Universitetsmuseum, UiT—The Arctic University of Norway, 9037 Tromsø, Norway; 3 Norwegian Institute for Nature Research (NINA), FRAM—High North Research Centre for Climate and the Environment, Langnes, P.O. Box 6606, 9296 Tromsø, Norway; 4 Applied Mathematics Department, University of Alicante, 03080 Alicante, Spain; 5 Department of Agriculture, Food, Environment, and Forestry (DAGRI), University of Florence, Piazzale Delle Cascine 18, 50144 Florence, Italy

**Keywords:** MODIS, NDVI, time series, onset of vegetation growth, trend, Arctic, Svalbard, spatial scales

## Abstract

The global temperature is increasing, and this is affecting the vegetation phenology in many parts of the world. The most prominent changes occur at northern latitudes such as our study area, which is Svalbard, located between 76°30′N and 80°50′N. A cloud-free time series of MODIS-NDVI data was processed. The dataset was interpolated to daily data during the 2000–2020 period with a 231.65 m pixel resolution. The onset of vegetation growth was mapped with a NDVI threshold method which corresponds well with a recent Sentinel-2 NDVI-based mapping of the onset of vegetation growth, which was in turn validated by a network of in-situ phenological data from time lapse cameras. The results show that the years 2000 and 2008 were extreme in terms of the late onset of vegetation growth. The year 2020 had the earliest onset of vegetation growth on Svalbard during the 21-year study. Each year since 2013 had an earlier or equally early timing in terms of the onset of the growth season compared with the 2000–2020 average. A linear trend of 0.57 days per year resulted in an earlier onset of growth of 12 days on average for the entire archipelago of Svalbard in 2020 compared to 2000.

## 1 Introduction

Climate change is shifting the phenological cycles of plants, thereby altering the function of ecosystems, which in turn induces feedbacks to climate systems [1]. The global temperature is increasing, and this is affecting the vegetation phenology in many parts of the world, with most prominent changes occurring in northern latitudes. The Arctic is a region that is expected to experience a high increase in temperature [2,3]. A recent study [4] shows that exceptional warming occurred over the northern Barents area due to a strong decline in sea ice concentration. The authors reported a temperature increase of up to 2.7 °C per decade annually at the isolated island Karl XII-øya, located in the northeastern part of the Svalbard archipelago. This warming is unprecedented in this region and exceptional in the Arctic and even on a global scale. The increase is highest during autumn and lowest during summer.

At northern latitudes, time series of satellite data show significant advances in the onset of vegetation growth and increased greening (in terms of peak NDVI or time-integrated NDVI) in recent decades [5–9]. During the 1982–2015 period, the greening trend was most pronounced in the continental parts of the High Arctic, while oceanic to moderately continental regions, including the high Arctic islands of Svalbard, showed weaker trends [7]. However, trends in NDVI data produced from different satellite datasets or using different methods do not always correspond at a given location [10,11]. Guay et al. [12] compared different but widely used global/continental NDVI datasets and found equally large areas of agreement (40%) and disagreement (40%) between the Global Inventory Modeling and Mapping Studies (GIMMS) NDVI datasets as well as with more modern datasets. Thus, it can be challenging to distinguish ecological changes from differences due to methods and sensor/platform-related issues when interpreting localized spectral greening signals [13]. The ecological processes underlying spectral parameters measured by satellites are diverse and may unfold across overlapping scales, extents, and time frames. Hence, there is clearly a need for further regional studies at different spatial scales integrated with in-situ data, which could also be used for validating more continental/global datasets. In particular, this is important at very high latitudes, where optical satellite sensors have a limited annual window because of the prolonged polar night and low sun angles and persistent cloud cover reduce data quality during the summer season [13].

The onset of the vegetation growing season on Svalbard was mapped for the 2000–2013 period using remotely sensed data from the spaceborne Moderate Resolution Imaging Spectroradiometer (MODIS) onboard the spacecraft Terra [14]. Overall, that study found no significant trends in the onset of the growing season during the 2000–2013 period. Since then, Svalbard has experienced temperature increases in all months compared to 2000–2013 [15]. Recently, a cloud-free time series of Sentinel-2 NDVI data was processed and used to map the onset of vegetation growth in central Svalbard during the 2016–2019 period [16]. That study used in-situ phenological data from a network of time lapse cameras designed for upscaling to the 10 m pixel resolution Sentinel-2 data.

In the present study, we process an entirely new dataset from the MODIS Collection 6, removing the clouds and interpolating to daily data, for the 2000–2020 period with a 231.65 m pixel resolution. To bridge the spatial gap, we use the new MODIS dataset to map the onset of growth with a method that corresponds well with the 10 m resolution Sentinel-2-based onset of growth.

The exploitation of the wide time range derived from the MODIS time series together with the finer spatial scale of Sentinel-2 as validation in addition to ground truth thus offers a novel opportunity to understand the trend patterns of the onset of the growing season in Svalbard.

## 2 Materials and Methods

### 2.1 Study Area

**Figure 1:**
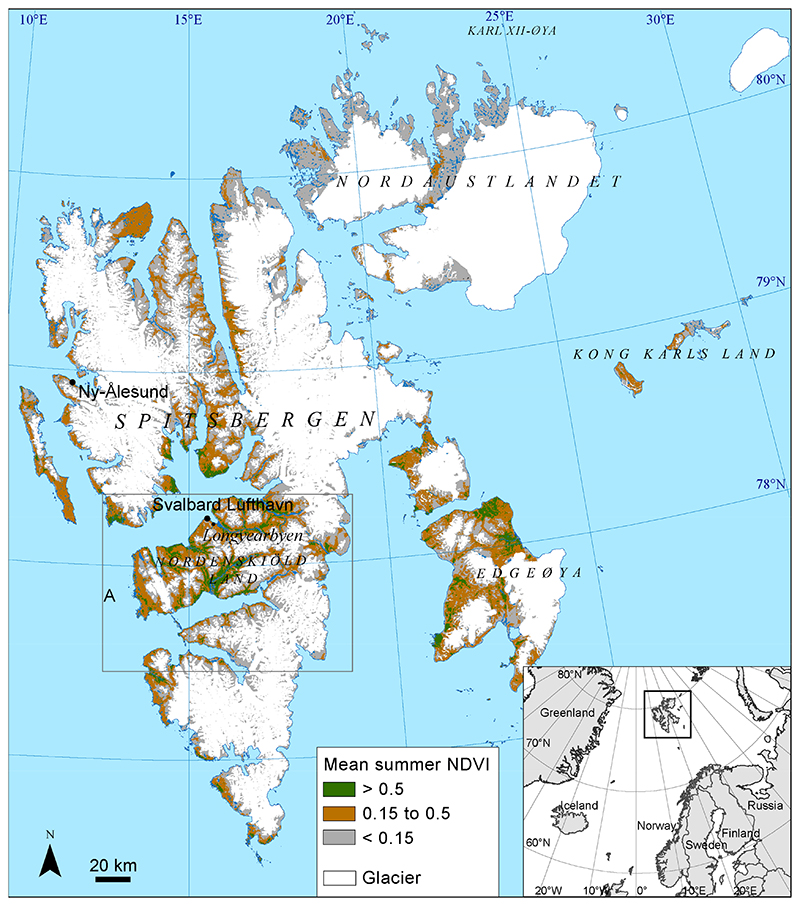
The study area of Svalbard, showing mean NDVI values for the 25 July to 1 August period (MODIS Terra 21-year, mean 2000–2020), and the location of the meteorological stations Svalbard Lufthavn and Ny-Ålesund. Glaciers are from the Svalbard S250 dataset. ‘A’ indicates the area recently mapped by using time series of Sentinel-2 data [16].

Svalbard is located between 76°30′–80°50′N and 10°30′–34°45′E. The main island of the archipelago is Spitsbergen, followed by Nordaustlandet and Edgeøya (Figure 1), and the total area is approximately 62,000 km^2^. Glaciers dominate in most of the islands and cover more than half of the land surface. The largest ice-free areas with vegetation are found on the Nordenskiöld Land peninsula in central Spitsbergen [17]. This is also the area with the highest summer temperatures.

The meteorological station Svalbard Lufthavn, close to the administration center Longyearbyen (Figure 1), shows a mean July air temperature of 7.36 ° C during 2000–2020 (Table 1, Table S1). The temperatures have increased for all months during the 2000–2020 period. For July, a linear trend shows an increase in temperature of 0.09 ° C per year, corresponding to 1.83 °C for the 21-year period. The annual mean temperature has increased even more, with a temperature increase of 0.12 °C per year or 2.55 °C for the whole period. For the meteorological station Ny-Ålesund in the northwestern part of Svalbard (Figure 1), the temperature increase is slightly lower for June and July as compared with Svalbard Lufthavn but higher in May and August (Table 1).

### 2.2 Processing MODIS Data—Cloud Removal and Interpolation

Two MODIS Terra products, both with 8-day temporal composites, were used. The MOD09A1.V006 product, with surface reflectance values for bands 1–7 at a 463.31 m pixel resolution, was used to extract information about the cloud cover, as was MOD09Q1.The V006 product, with surface values for bands 1 and 2 at a 231.65 m pixel resolution, was used to calculate NDVI values. Both datasets were processed for the 2000–2020 period and reprojected to the ETRS89 Zone 33N (EPSG: 25833) projection. Different quality assessment (QA) values in the dataset indicate cloud cover, and the exact day of acquisition is recorded in the dataset for each pixel. For each 8-day period, we visually evaluated State QA values and own cloud masks. In most cases, one of the State QA values or a combination of the State QA values or certain own masks detected the clouds in the data covering Svalbard. However, in a small number of cases, none of the State QA values or our own cloud algorithms were able to detect the noise in the data. In these cases, we manually masked out the noisy parts by drawing polygons around them. The method is further described in Karlsen et al. [14]. This is time-consuming work, but it is only done once and ensures that most of the noise in the datasets is removed and that most of the noise-free data is retained. Then, NDVI values were calculated for the cloud-free pixels from the MOD09Q1.V006 dataset at 231.65 m resolution. For each 8-day period, the acquisition day of each pixel is known, and this information was used in gap filling to daily data. The gap-filling to daily data was achieved by performing the kernel ridge regression (KRR) machine learning method [18], with the data then being smoothed with a Savitzky–Golay filter (5 in windows size and 1 iteration), using the Decomposition and Analysis of Time Series Software (DATimeS) [19]. Several interpolation methods were tried on the cloud-free NDVI data. The KRR method was chosen as it performs well overall, and the goodness of fit statistic shows the root mean square error on 0.007 in NDVI values (mean 2000–2020).

Areas with mean NDVI < 0.15 (mean 2000–2020) for the 25 July to 1 August period (Figure 1) were not included in the dataset as they have no or at most very sparse vegetation cover. The resulting dataset is daily clear-sky NDVI-maps with 231.65 m resolution for the 2000–2020 period.

### 2.3 Phenological In-Situ Data and Time Series of Sentinel-2 Data

Phenological observations designed to be upscaled by Sentinel-2 data have been established in the Adventdalen and adjacent Endalen valleys close to Longyearbyen. The phenological observations were carried out with time lapse cameras on dominant/sub-dominant vascular plants species within seven homogeneous sites/vegetation types. The smallest of these homogeneous sites/vegetation types is about 1400 m^2^ and the others range from 7300 to 36,100 m^2^. The phenophases recorded from the dwarf shrubs are ‘>5 leaves unfolded, but not yet full size’, and the corresponding phenophase of the graminoids were ‘>5 leaves (>3 cm) clearly visible’. This phenological stage is defined as the ‘onset of growth’ in this study. It happens rapidly, is easily observed in the field on Svalbard, and is extracted from phenocams with an inaccuracy of less than 4 days. More details regarding the observations and observation sites are found in Karlsen et al. [16].

Recently, a cloud-free time series of daily Sentinel-2 data for the 2016–2019 period was processed [16]. This dataset covers the central parts of Svalbard (outlined in Figure 1) and was used in mapping the onset of vegetation growth validated from the described in-situ phenological data. For the years 2016–2018, the dataset had several temporal gaps due to frequent cloud cover, whereas the 2019 season had few time series gaps, defined as pixels with more than 10 days elapse since the last cloud-free day.

### 2.4 Mapping the Onset of Vegetation Growth

When Karlsen et al. [16] used time series of Sentinel-2 NDVI data to map the onset of vegetation growth in central Svalbard, they first computed the 4-year (2016–2019) mean NDVI value for every pixel in the study area for the 10 July to 5 August period. This period was chosen as the period where the leaves are fully open and before senescence starts and reduced the “noise” from snow-covered ground. The onset of vegetation growth at each pixel each year was defined as the time when the NDVI value each year exceeded 69% of the 10 July to 5 August 4-year mean NDVI value. This threshold gave high and significant correlation (r^2^ = 0.47, *n* = 38, p < 0.0001) with the onset of growth observed in the field with time lapse cameras across seven vegetation types, and on average, the NDVI-based onset of growth occurs one day later than the phenocam-based growth (day of year 171 vs. 170, 19 vs. 20 June). The largest bias and variability between the phenocam and the Sentinel-2 NDVI-based onset of growth were found in the moss tundra, where the scattered occurrences of vascular plants only contribute very little to the NDVI signal. In the moss tundra, the phenocam-observed onset of growth in vascular plants occurs up to 14 days earlier and up to 5 days later compared with the Sentinel-2 NDVI-based onset.

In the present study, we use the Sentinel-2 NDVI-based onset of vegetation growth map of central Svalbard from the year 2019 as validation for the MODIS based mapping. We only use the Sentinel-2 NDVI-based onset from the year 2019 in the comparison with MODIS data, this since the years 2016 and 2017 only have data from the Sentinel-2A satellite (also from Sentinel-2B after July 2017), and we did not use the onset from 2018 due to very frequent cloud cover that season, resulting in data gaps in the Sentinel-2 time series that year. First, the pixel resolution in the Sentinel-2 based onset of vegetation growth map was resampled to the coarser MODIS resolution. This was achieved by aggregating the mean of 23 × 23 Sentinel-2 pixels from the original 10 m to 230 m. Next, we compared the onset maps by subtracting the Sentinel-2-based onset with the MODIS based onset.

Then, the MODIS based onset of growth at each pixel each year was defined as the time when the NDVI value each year exceeded 63% of the 10 July to 5 August 21-year (2000–2020) mean NDVI value. This NDVI threshold gave the best fit with the Sentinel-2-based mapping of the onset of growth in 2019. The threshold in MODIS-NDVI data is lower than in Sentinel-2 NDVI data due to different bandwidths in red and near infra-red bands in Sentinel-2 vs. MODIS, resulting in differences in NDVI value between the sensors.

## 3 Results

### 3.1 Comparing Sentinel-2- and MODIS-Based Mapping of Onset of Growth

**Figure 2:**
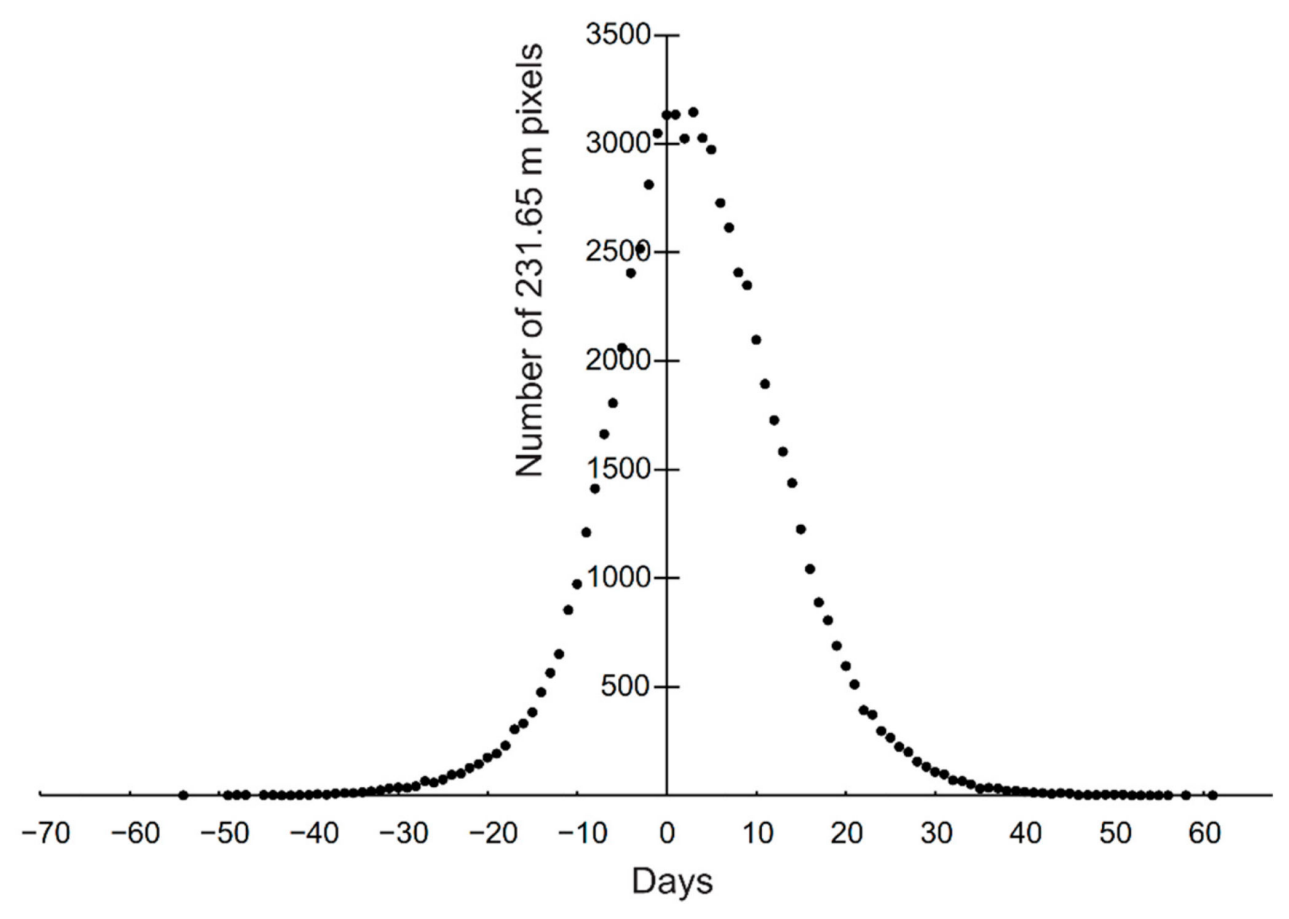
Comparison between Sentinel-2- and MODIS-based mapped day of onset of vegetation growth in 2019 in central Svalbard. The figure shows number of pixels and number of days differences between the Sentinel-2-based and the MODIS-based day of onset of growth after the Sentinel-2 pixels were resampled to MODIS pixel size.

**Figure 3:**
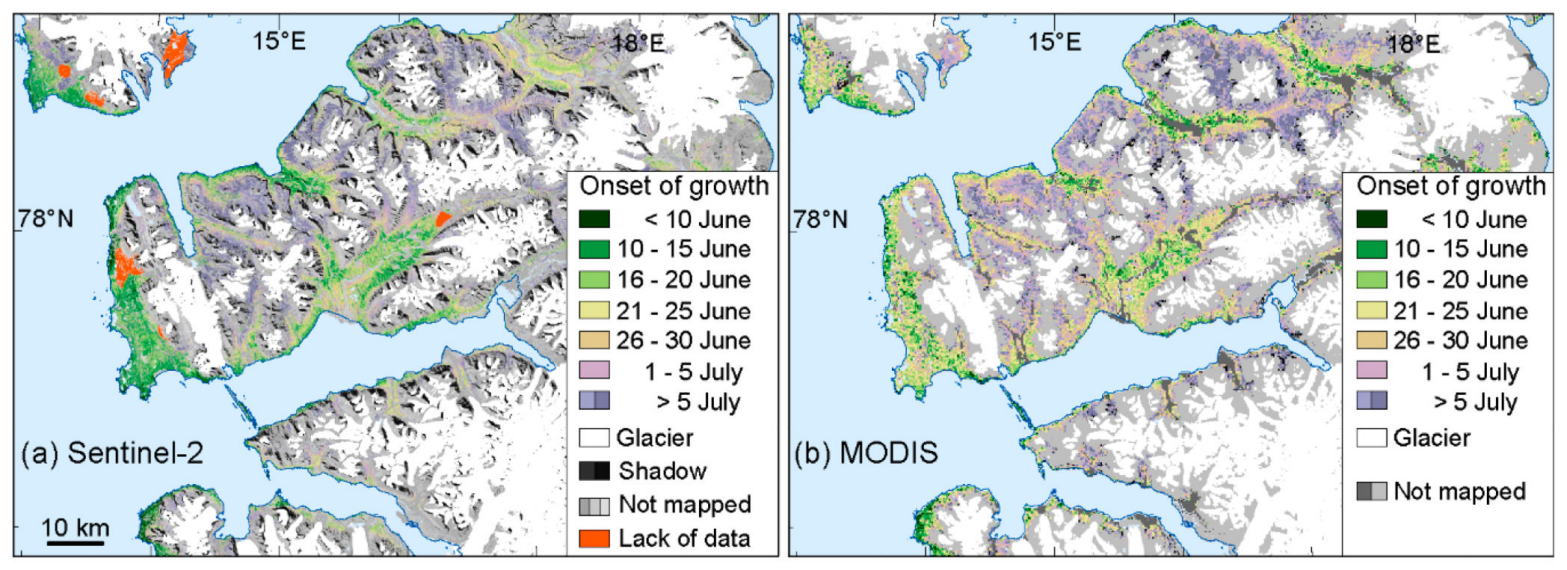
Onset of vegetation growth in 2019 for central Svalbard. Mapped with (a) time series of cloud-free Sentinel-2 NDVI data (extracted from Karlsen et al. [16]) and (b) mapped with time series of MODIS NDVI data.

Figure 2 shows a comparison of day of onset of vegetation growth from Sentinel-2 minus MODIS. Despite the original sensors having very different spatial scales, most of the pixels (71%) had a disagreement between the products of 10 days or less (Figure 2). The Sentinel-2-based map (with its original 10 m pixel resolution) (Figure 3a) and the MODIS based map (Figure 3b) show similar patterns in onset of vegetation growth, except in the most topographically complex areas, where the different spatial scales create disagreements. Additionally, on the west coast, the maps indicate a slightly earlier onset on the Sentinel-2-based map (Figure 3a) compared with the MODIS-based map (Figure 3b). This might be the result of the large difference in spatial resolution (10 m versus 231.65 m), which results in a better detection of the onset by Sentinel-2 compared to MODIS.

### 3.2 Variability and Trends

**Figure 4:**
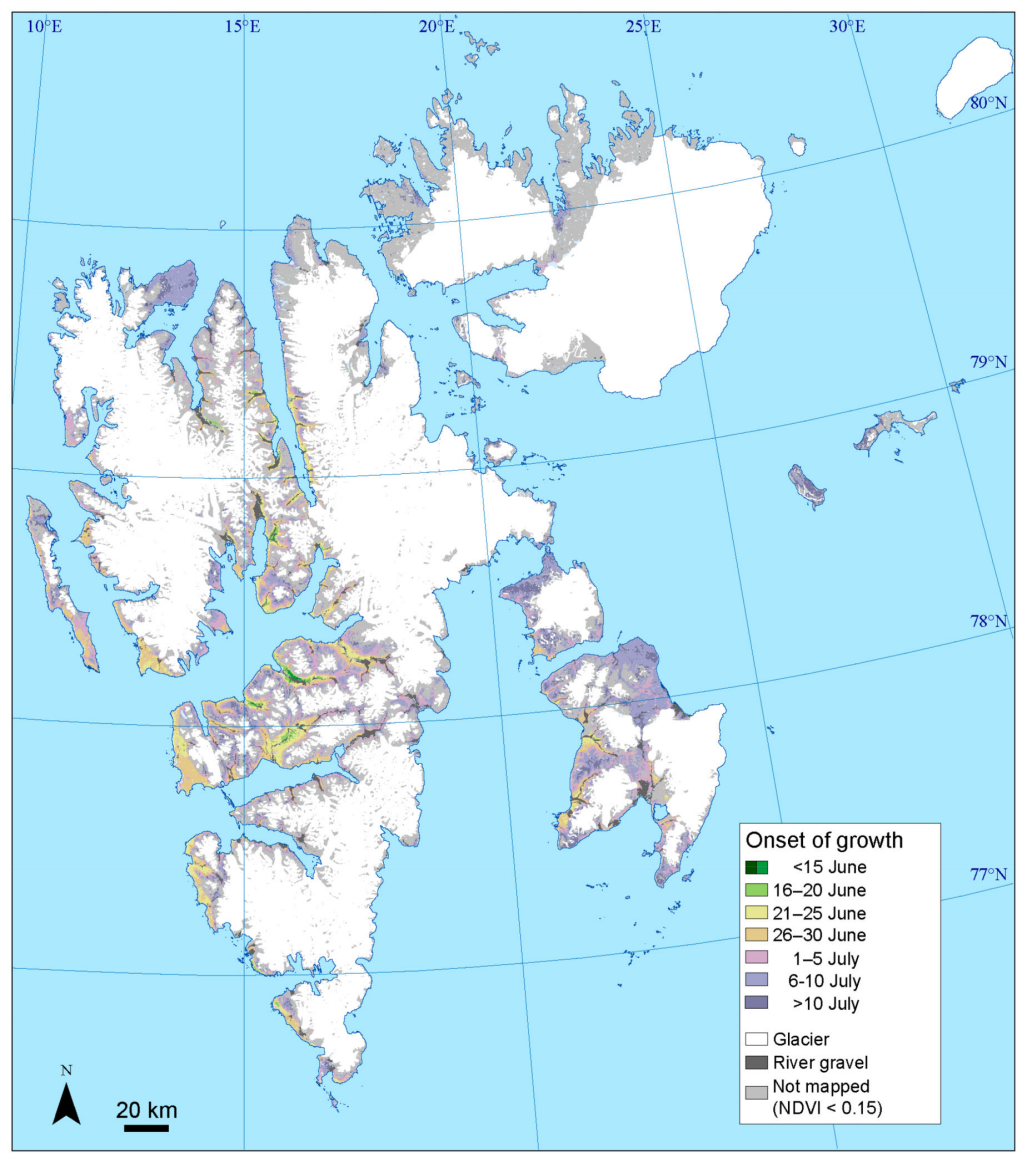
Mean (2000–2020) onset of vegetation growth.

The mean onset of growth (2000–2020) occurred before 16 June in only 0.1% (12 km^2^) of the mapped area (Figure 4), with these areas being certain spots in the main valleys on Nordenskiöld Land. During the latter part of June (16–30 June), the onset of growth occurred in 25.3% (2010 km^2^) of the mapped area, located mainly in the lowland of central Spitsbergen. In most of the mapped area, the onset occurred between 1 and 10 July, (57.7%, 4584 km^2^). This category occurred in most of the eastern and northern parts of the archipelago and at higher altitudes in western and central parts of Spitsbergen. A late average onset, after 10 July, occurred in 16.9% (1341 km^2^) of mapped areas.

**Figure 5:**
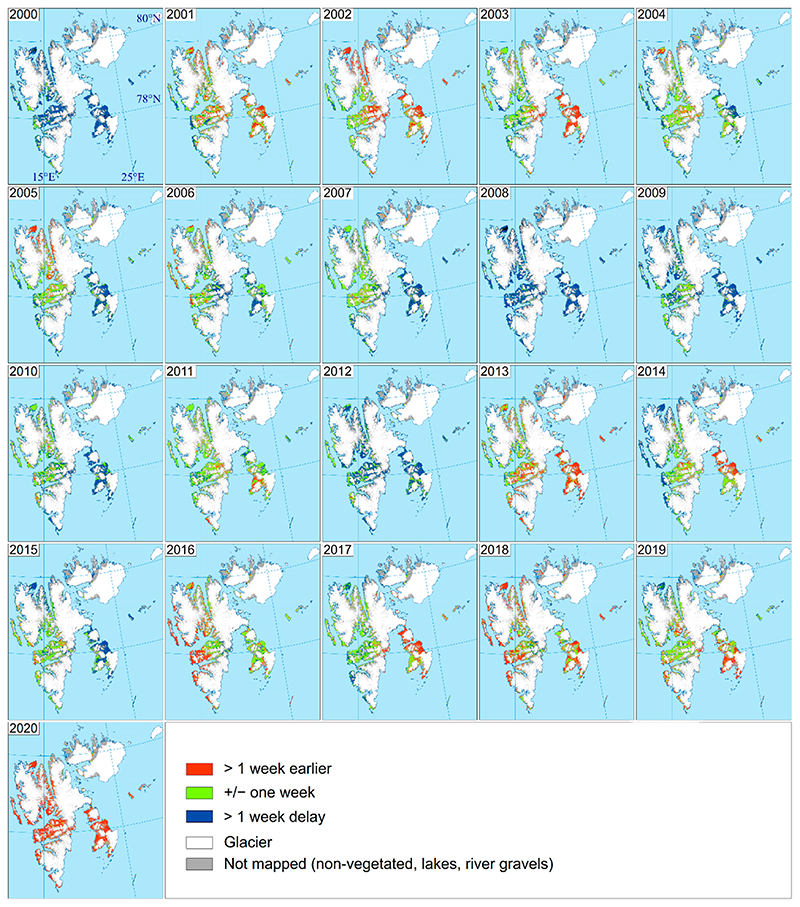
Regional variation in onset of vegetation growth as compared with 2000–2020 mean values.

**Figure 6:**
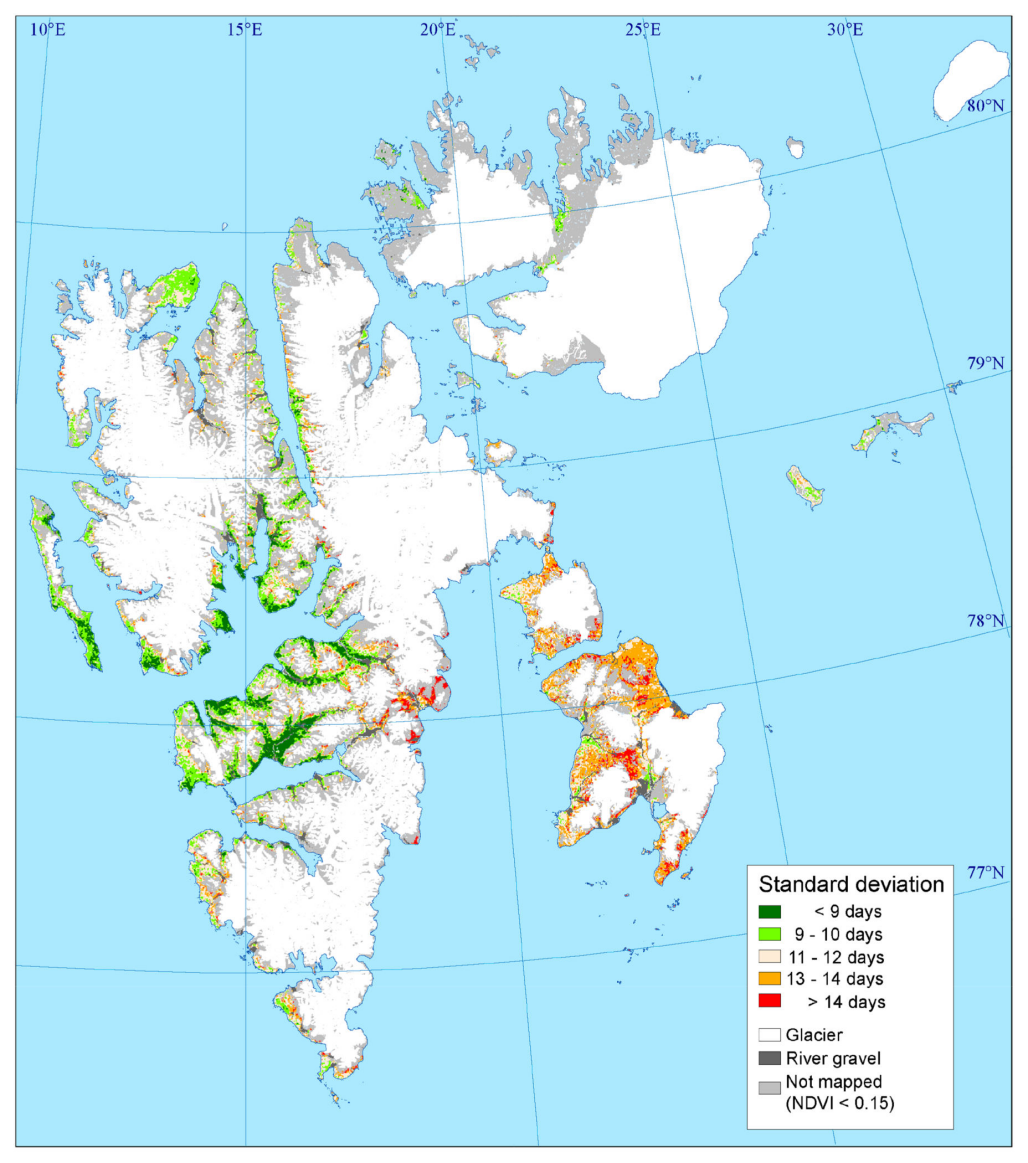
Standard deviation in onset of vegetation growth for the 2000–2020 period.

The years 2000 and 2008 were extreme in terms of a late onset of vegetation growth (Figure 5), and during those years, 84% and 89% of the mapped area shows a more than one-week later onset of growth compared to the 2000–2020 average, respectively. In 2008, only 7.6% of the mapped area had an onset of growth before 1 July. Furthermore, 2009 had a late onset, except for most of Nordenskiöld Land. The most extreme in terms of early onset was the year 2020. During this year, 91% of the mapped area had more than a one-week earlier onset than the 21-year average, with the remaining 9%, i.e., areas mainly found in the valleys Adventdalen and Reindalen on Nordenskiöld Land, having average timing. This year, the onset of growth occurred before 16 June in 42.4% of the mapped area. Other years with early onset (>47% of the mapped area had >1 week earlier than 2000–2020 mean value) were 2002, 2013, 2016, and 2018. The largest variability is shown for Edgeøya, where onset was either early or late and only the year 2016 was a typical average year. The standard deviation for the onset of growth for the mapping period was mostly more than 13 days in these eastern parts (Figure 6). While the large valley (Adventdalen and Reindalen) on Nordenskiöld Land shows less variability between the years, about 13 of the 21 years were classified as close to mean in these valleys, with the standard deviations being 9 days or less.

**Figure 7:**
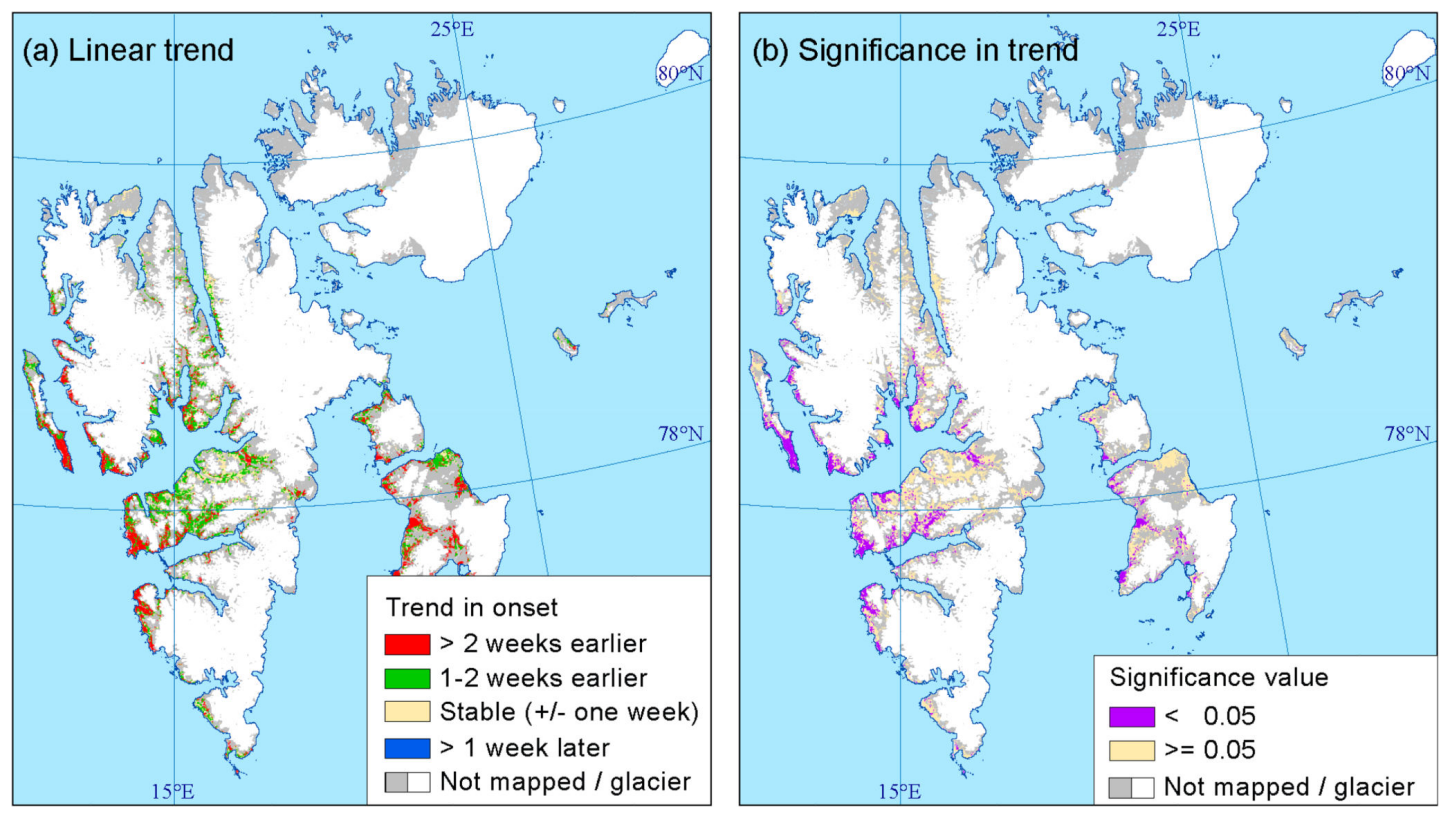
(a) Number of days of the earlier start of onset of vegetation growth in 2020, as compared to 2000, assuming a linear trend. (b) Areas where the linear trend is significant (p < 0.05).

Figure 7a shows the 21-year linear trend in onset of vegetation growth. On average for the entire archipelago, the trend was 12 days (0.57 days pr year) earlier during the 2000 to 2020 period. However, there are regional differences. On the west coast and for parts of Edgeøya island, there was a trend of more than two weeks earlier onset, with these areas covering 36% of the mapped areas. Most of these linear trends are also significant (p < 0.05) (Figure 7b), with some exceptions, such as the eastern parts of Edgeøya island, which has large variability between the years (Figure 5 and 6). A trend of 1 to 2 weeks earlier onset occurred in 42% of the mapped areas and was found in most parts of the archipelago. Less dramatic changes (+/− one week) in onset of growth occurred in 22% of the area, and this was found in most of Adventdalen valley and parts of the Reindalen and Colesdalen valleys on Nordenskiöld Land. Only 0.3% of the mapped area had a linear trend, indicating a later onset of vegetation growth.

## 4 Discussion

### 4.1 Changes in Onset of Vegetation Growth

The annual temperature has significantly increased on Svalbard during the 2000 to 2020 period (Table 1, Table S1). A previous MODIS-based mapping of the onset of growth for the 2000–2013 period [14] found no significant trend for that period due to no significant increase in June–July temperatures, which is the main driver of the onset of growth on the MODIS scale [20]. Increases in temperatures prior to 2013 have been measured in the other months, especially during autumn and winter [3,15], which do not directly influence the start or timing of the onset of the growing season. On the contrary, years such as 2000, 2008, 2009, 2010, 2012, and 2015 showed a later onset of vegetation growth. The later onset for the years 2010, 2012, and 2015 could also be due to milder winters with frequent “rain on snow events” during winter causing thick ice layers in the snowpack [3,21]. This resulted in a later melting of snow and ice, resulting in browning and damage/injury to different plant species [22], which in turn led to a later start and peak in terms of the growing season [7,23]. Furthermore, our result shows that four of the five earliest years (early onset) during the 2000–2020 period have been since 2013 (2013, 2016, 2018, and 2020). During the first decade (2000–2010), it was only in the year 2002 that more than 50% of the mapped area was classified as early onset. In fact, all years since 2013 have had an early or average timing in terms of the onset of vegetation growth, with certain local exceptions (in 2014, the westernmost and northernmost coast of Spitsbergen had late onset, and in 2015, northeasternmost Edgeøya and northernmost Svalbard had late onset). The year 2020 appears to be the earliest year among all concerning the onset of growth on Svalbard during the 21-year period. This year, most of Svalbard had a much earlier onset of growth compared with the mean values, except the Longyearbyen area and other parts of Nordenskiöld Land, where values were only slightly earlier. The winter 2019/20 was normal and cold on Svalbard, with normal (dry) and thin snow layers resulting in an earlier snow melt for large parts of Svalbard [15]. The large increase in temperature recently reported for the northeastern parts of Svalbard [4] is not clearly visible in the present since most of the northeastern parts of the Svalbard archipelago are masked out as ‘not mapped (NDVI < 0.15)’.

### 4.2 Closing the Spatial and Temporal Gaps

The present study is based on measurements of the onset of vegetation growth on several spatial scales. At plot scale, the onset of growth was extracted from 5 × 5 cm^2^ areas in time lapse images of a dominating vascular plant, representing large (>7300 m^2^) homogenous areas. This was used to validate the Sentinel-2-based mapping of onset based on 10 m pixels resolution [16], and finally the MODIS based mapping of onset with 231.65m pixels resolution was compared with the Sentinel-2-based mapping of onset in the present study. The temporal scales were harmonized to daily data, where time lapse cameras capture several photos pr day, for extracting the day of onset of the growth of both shrubs and graminoids. Both the Sentinel-2 and MODIS data were interpolated to daily data after removing clouds in order to map the day of onset.

Ecological changes occur more rapidly on local scales compared with regional scales [24]. For instance, in one of the in-situ observation sites, a *Dupontia fisheri*-dominated mire, all the individual plants we observed using a time lapse camera, as well as most of the homogeneous area it represented, was heavily grazed by barnacle geese (*Branta leucopsis*) in 2017, and the cover of *Dupontia fisheri* at peak growth decreases from 87% to 48%. Still, it was possible to detect the onset of growth the following year using time lapse cameras. However, this grazing lowered the NDVI value in several Sentinel-2 10 m pixels and thereby the timing of the onset of growth was mapped too late. However, the grazing gave hardly any spectral signal in the MODIS 231.65 m pixel resolution and likely did not significantly influence the MODIS-based mapping of the onset of growth, illustrating that the mapped onset is scale-dependent and that mechanistic links between the scales are complex and difficult to understand without ground data. Hence, the influence of this grazing effect as well as other biotic and abiotic disturbances across the spatial scales mostly remain unclear. This is illustrated when comparing pixel-by-pixel MODIS- and Sentinel-2-based mapping of onset. Although most pixels show close to the same day of onset (after Sentinel-2 was resampled to the 231.65 m MODIS pixel size, Figure 2), 29% of the pixels still had a difference of more than 10 days earlier/later regarding the mapping of the onset of vegetation growth. It is reasonable to believe that the local topography influences the NDVI value in MODIS more than in Sentinel-2. Where MODIS pixels include, for instance, patches of snow, standing water in mires, and possibly certain shadows due to the low sun angle, such area are excluded by the Sentinel-2-based mapping.

Another main uncertainty in the mapping of onset could arise moss tundra, as bryophytes have a phenological cycle that differ from vascular plants and is more related to moisture than temperatures. For instance, the widespread *Tomentypnum nitens* get mature capsules in early–mid summer. Hence, a separate definition of growth season stages should be defined for moss tundra. Karlsen et al. [16] found a large bias between the Sentinel-2 NDVI-based mapped onset of growth and the phenocam-based onset of growth in a moss tundra of the widespread *Aulacomnium turgidum-Tomentypnum nitens* type. May et al. [25] showed that high moisture content gave high NDVI values in Arctic moss communities, as high moisture lowers the reflectance value in the red band more than in the NIR band, which gives high NDVI values. Since moss tundra is widespread on Svalbard [17] and patches of moss tundra would occur in most of the pixels when aggregating vegetation types to MODIS pixels, this would create uncertainty in the MODIS-based mapping but would be more easily detected in Sentinel-2, where most of the plant communities are identified. Hence, when aggregating from Sentinel-2 to MODIS scale, the results quantify large effects in terms of landscape heterogeneity. Overall, the scale gap between the onset of growth observed in-situ in plant communities and the onset mapped with Sentinel-2 data appears to be relatively easier to understand compared to the spatial scale gap between Sentinel-2 and MODIS. It is reasonable to believe that the spatial pattern and year-to-year differences in the onset of vegetation growth is trustable on the MODIS scale, but the actual date must be interpreted with care, mainly due to uncertainties related to landscape heterogeneity and patches of moss tundra.

## 5 Conclusions

To map the onset of vegetation growth in Svalbard, the present study processed and used 21 years (2000–2020) of cloud-free MODIS NDVI data with a 231.65 m pixel resolution. Previously, a Sentinel-2 NDVI-based mapping of the onset of vegetation growth in 2019, with a 10 m pixel resolution and thoroughly validated by the use of in-situ time lapse cameras was carried for central Svalbard. Comparing the MODIS-NDVI and Sentinel-2 NDVI-based mapping shows that the majority of the pixels had almost identical days of onset; however, 29% of the pixels still had a deviation of more than 10 days. This disagreement might be a result of the large difference in spatial resolution (10 vs. 231.65 m) which results in better onset detection by Sentinel-2 compared to MODIS. It is reasonable to believe that the local topography influences the NDVI value of MODIS more than that of Sentinel-2. Where MODIS pixels include, for instance, patches of snow, standing water in mires, and possibly certain shadows due to the low sun angle, these are mostly excluded by the Sentinel-2-based mapping. The present MODIS-NDVI-based mapping reveals large spatial variability within years and between years in the onset of vegetation growth. The year 2020 appears to have been the earliest onset of growth on Svalbard during the 21-year (2000–2020) study period. Each year since 2013 had earlier or equally early timings compared with the 2000–2020 mean value. The trend of earlier onset was most pronounced and significant (p < 0.05) along the west coast of Spitsbergen and in parts of the island Edgeøya in the eastern parts of the archipelago.

## Figures and Tables

**Table 1:** Monthly and mean annual temperature (°C) for the two meteorological stations Svalbard Lufthavn and Ny-Ålesund based on data from the Norwegian Centre for Climate Services [15]. Temperatures for the period 2000–2020 were analyzed, and, assuming a linear trend, ** *p* < 0.01, * *p* < 0.05.

Station Number and Name	May	June	July	August	Annual
99840 Svalbard Lufthavn					
Mean	−1.53	3.99	7.36	6.33	−3.15
Linear trend	2.27	1.65 **	1.83 **	0.44	2.55 **
99910 Ny-Ålesund					
Mean	−1.95	2.96	6.08	4.76	–3.61
Linear trend	2.49 *	1.52 *	1.61 **	0.76	2.18 **

## Data Availability

Not applicable.

## Footnotes

**Supplementary Materials:** The following supporting information can be downloaded at: https://www.mdpi.com/article/10.3390/rs14246346/s1, Table S1: Monthly and mean annual temperature.
